# New Structural and Mechanistic Insights Into Functional Roles of Cytochrome *b*_559_ in Photosystem II

**DOI:** 10.3389/fpls.2022.914922

**Published:** 2022-06-08

**Authors:** Yi-Fang Chiu, Hsiu-An Chu

**Affiliations:** Institute of Plant and Microbial Biology, Academia Sinica, Taipei, Taiwan

**Keywords:** photosynthesis, photosystem II, cytochrome *b*_559_, site-directed mutagenesis, photoprotection, photoinhibition

## Abstract

Cytochrome (Cyt) *b*_559_ is a key component of the photosystem II (PSII) complex for its assembly and proper function. Previous studies have suggested that Cyt*b*_559_ has functional roles in early assembly of PSII and in secondary electron transfer pathways that protect PSII against photoinhibition. In addition, the Cyt*b*_559_ in various PSII preparations exhibited multiple different redox potential forms. However, the precise functional roles of Cyt*b*_559_ in PSII remain unclear. Recent site-directed mutagenesis studies combined with functional genomics and biochemical analysis, as well as high-resolution x-ray crystallography and cryo-electron microscopy studies on native, inactive, and assembly intermediates of PSII have provided important new structural and mechanistic insights into the functional roles of Cyt*b*_559_. This mini-review gives an overview of new exciting results and their significance for understanding the structural and functional roles of Cyt*b*_559_ in PSII.

## Introduction

Cytochrome (Cyt) *b*_559_ is an essential component of the photosystem II (PSII) complex for proper functioning and assembly (reviews by Whitmarsh and Pakrasi, 1996; Stewart and Brudvig, 1998; Shinopoulos and Brudvig, 2012; Müh and Zouni, 2015; Chu and Chiu, 2016). Cyt*b*_559_ is present in the PSII of all oxygenic photosynthetic organisms but is not found in anoxygenic type II reaction centers of photosynthetic bacteria (Majumder and Blankenship, 2015). Thus, Cyt*b*_559_ likely co-evolved with the oxygen-evolving function of cyanobacteria. Cyt*b*_559_ is a heme-bridged heterodimer protein that is comprised of 1 α and 1 β subunit (subunits PsbE and PsbF encoded by *psbE* and *psbF*, respectively) (Umena et al., 2011; review by Müh and Zouni, 2015). Each subunit provides a histidine ligand for the non-covalently bound heme, which is located near the cytoplasmic side of PSII (Babcock et al., 1985). In contrast, most mono-heme cytochromes are made of a single polypeptide (Majumder and Blankenship, 2015). In addition, the Cyt*b*_559_ in different PSII preparations features multiple distinct redox potential forms: high potential (HP) with Em + 370–400 mV, intermediate potential (IP) with Em of about 200 mV, and low potential (LP) with Em of about 0–80 mV (Ortega et al., 1988; Thompson et al., 1989; Kaminskaya et al., 1999; Roncel et al., 2001). The redox potential of the HP form in Cyt*b*_559_ is unusually high for *b*-type cytochromes. The redox midpoint potentials of most *b*-type cytochromes were in the range of −225 to +168 mV (Liu et al., 2014). The HP form is typically predominant in native PSII preparations, whereas the IP and LP forms are predominant in less intact or inactive PSII preparations such as Tris-washing treatment, which removes manganese and extrinsic proteins of PSII (Ghanotakis et al., 1986; Thompson et al., 1989; Kaminskaya et al., 1999; Roncel et al., 2003).

Many studies have suggested that Cyt*b*_559_ may participate in secondary electron transfer pathways that protect PSII against photoinhibition (Heber et al., 1979; Thompson and Brudvig, 1988; Barber and De Las Rivas, 1993; Poulson et al., 1995; Faller et al., 2001; Tracewell and Brudvig, 2008; review by Shinopoulos and Brudvig, 2012). Cyt*b*_559_ in the HP form may donate its electron *via* a β-carotene molecule (CarD2) to reduce the highly oxidizing chlorophyll (P680^+^) in PSII reaction centers under donor-side photoinhibitory conditions. In addition, Cyt*b*_559_ may accept an electron from the acceptor side of PSII [e.g., ${\text{Q}}_{\text{B}}^{-}$ or reduced plastoquinones (PQs)] to prevent the formation of reactive oxygen species under acceptor-side photoinhibitory conditions (Nedbal et al., 1992; Barber and De Las Rivas, 1993; Bondarava et al., 2003, 2010). Moreover, previous studies showed that the Cyt*b*_559_ in tris-treated PSII has superoxide oxidase and reductase activities (Tiwari and Pospíšil, 2009; Pospisil, 2011). However, the precise functional roles of Cyt*b*_559_ in PSII are still not clear.

Previous mutagenesis studies on the model cyanobacterium *Synechocystis* sp. PCC 6803 (hereafter *Synechocystis*), the green alga *Chlamydomonas reinhardtii*, and tobacco (*Nicotiana tabacum*) all showed that the assembly of PSII reaction centers requires the presence of both the α and β subunits of Cyt*b*_559_ (Pakrasi et al., 1988; Morais et al., 1998; Swiatek et al., 2003). In addition, several studies demonstrated that Cyt*b*_559_ subunits interacted with D2 to form the essential intermediate complex D2 module during the early steps of PSII assembly (Komenda et al., 2004; Kiss et al., 2019). To study the structural and redox roles of the heme coordination of Cyt*b*_559_ in PSII, a series of site-directed mutants with mutations on histidine heme ligands of Cyt*b*_559_ was constructed and characterized in the model cyanobacterium *Synechocystis* and green alga *Chlamydomonas* (Pakrasi et al., 1991; Morais et al., 2001; Hung et al., 2007; Hamilton et al., 2014). Most of these Cyt*b*_559_ mutants accumulated only a little active PSII and, therefore, were unable to grow photoautotrophically. These previous findings suggest that proper coordination of the heme cofactor in Cyt*b*_559_ is important for the assembly or stability of PSII in *Synechocystis* (Pakrasi et al., 1991; Hung et al., 2007, 2010).

## Tandem Gene Amplification Restored PSII Accumulation of Cyt*b*_559_ Mutant Cyanobacteria

**Figure 1:**
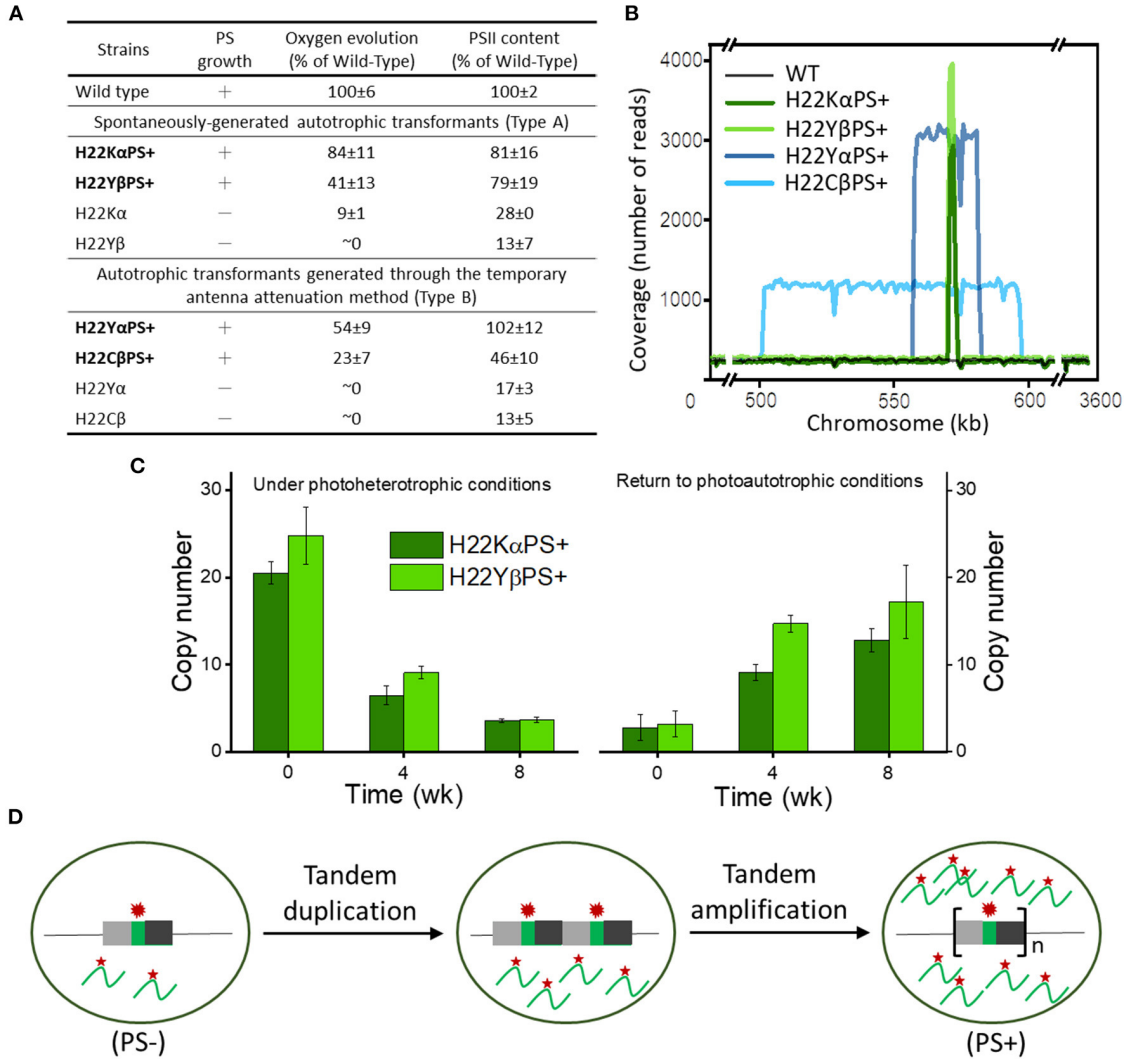
Photosynthetic growth and genetic properties of cytochrome *b*_559_ (Cyt*b*_559_) autotrophic transformants. **(A)** General properties of 2 distinct types of Cyt*b*_559_ autotrophic transformants. **(B)** Copy number, size, and location of repeat elements in autotrophic transformant cells. **(C)** Quantitative droplet digital polymerase chain reaction (PCR) analysis of the time course of copy number variation of *psbEFLJ* in autotrophic transformants grown under photoheterotrophic conditions and then returned to photoautotrophic conditions. **(D)** Model for tandem gene amplification of chromosome segments containing the *psbEFLJ* operon (asterisk) in autotrophic transformants recovering their photosynthetic growth [Reprinted with permission from Chiu et al. (2022), open access article by the New Phytologist Foundation].

A recent study developed a novel antenna attenuation method that restored photoautotrophic growth and PSII accumulation in several Cyt*b*_559_ mutant strains of *Synechocystis* with mutations in His-22 residues (heme ligands) of PsbE and PsbF (Figure 1A; Chiu et al., 2022). Whole-genome sequencing revealed that both types of autotrophic transformants (spontaneously generated in the early study or generated from the new antenna attenuation method in this recent study) carried 5–15 copies of tandem amplifications of chromosomal segments containing the mutated *psbEFLJ* operon (Figure 1B). Multiple copies of the *psbEFLJ* operon in these transformants were maintained only during autotrophic growth, whereas the number of copies gradually decreased under photoheterotrophic conditions (Figure 1C). This situation led to a 10- to 20-fold increase in transcript level of the mutated Cyt*b*_559_ gene (Figure 1D). The resulting overproduction of mutation-destabilized Cyt*b*_559_ subunits allowed for sufficient PSII accumulation and restored the photoautotrophic growth of the strains. This study demonstrated how tandem gene amplification restored PSII accumulation and photoautotrophic growth in Cyt*b*_559_ mutants of cyanobacteria, which may be an important adaptive mechanism of cyanobacteria for survival.

In contrast, in *Thermosynechococcus elongatus*, the heme coordination of Cyt*b*_559_ is not required for the assembly of PSII variants with psbA3 as the D1 subunit (Sugiura et al., 2015; Nakamura et al., 2019). Although the H23Aα and H23Mα Cyt*b*_559_ mutants of *T. elongatus* assembled only apo-Cyt*b*_559_ as unambiguously shown by electron paramagnetic resonance (EPR) analysis, they grew photoautotrophically and accumulated active PSII at the wild-type level (Sugiura et al., 2015). The greater structural stability of the thermophilic PSII complex is an important factor why heme ligand mutations do not significantly impair the PSII assembly in *T. elongatus*.

## Structural Determinants of Redox Potentials of Cyt*b*_559_

One of the distinct features of Cyt*b*_559_ in PSII is the presence of different redox potential forms. The HP form of Cyt*b*_559_ predominates in native PSII preparations of plants and *Thermosynechococcus*. In addition, for some unknown reason, intact PSII preparations from *Synechocystis* contained primarily the IP form of Cyt*b*_559_ but lacked the HP form (Ortega et al., 1994; Chiu et al., 2009). Under Tris-washing treatments, inactive PSII preparations from plants and *Synechocystis* usually contained predominantly the LP form (Thompson et al., 1989; Berthomieu et al., 1992; Mamedov et al., 2007; Chiu et al., 2009), whereas inactive PSII preparations from *Thermosynechococcus* contained primarily the IP form and lacked the LP form (Roncel et al., 2003). Therefore, the redox properties of Cyt*b*_559_ in PSII significantly differ in different species.

**Figure 2:**
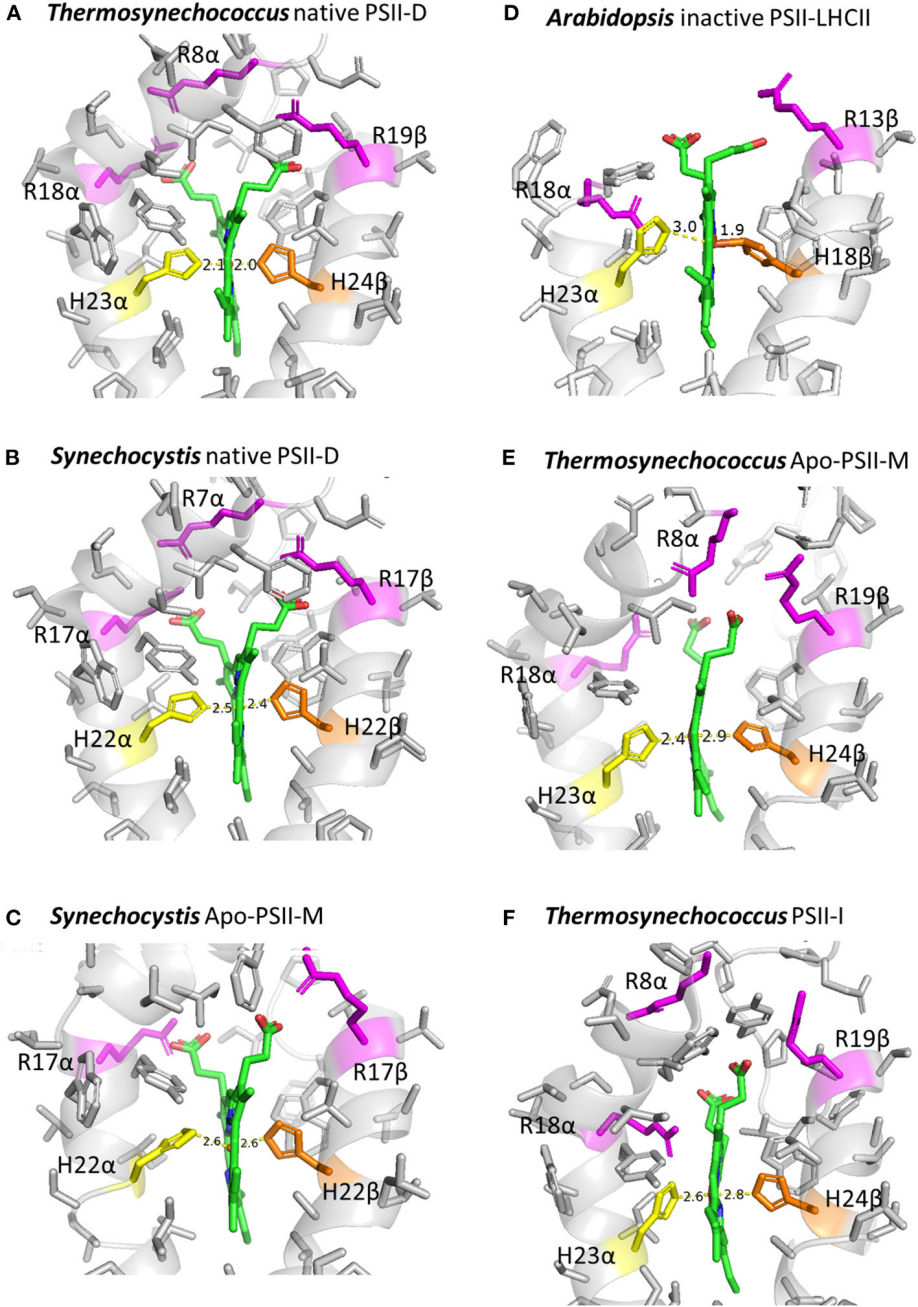
Heme coordination environments of Cyt*b*_559_ in cryo-EM structural models of different types of PSII preparations. **(A)** Native PSII dimer (PSII-D) of *Thermosynechococcus* (PDB 7D1U); **(B)** native PSII-D of *Synechocystis* (PDB 7RCV); **(C)** inactive PSII monomer (Apo-PSII-M) of *Synechocystis* (PDB 6WJ6); **(D)** inactive PSII-LHCII supercomplex from *Arabidopsis* (PDB 7OUI); **(E)** Apo-PSII-M of *Thermosynechococcus* (PDB 7NHO); and **(F)** PSII assembly intermediate (PSII-I) of *Thermosynechococcus* (PDB 7NHP). The figures were created using PyMol.

Structural determinants of the different redox-potential forms of Cyt*b*_559_ are still not clear. Previous studies suggested that the different redox-potential forms may be due to changes in hydrophobicity of the heme ligation environment (Krishtalik et al., 1993; Roncel et al., 2003), mutual orientation of the planes of histidine heme ligands (Babcock et al., 1985), or protonation or H-bonding pattern of the heme ligation environment (Ortega et al., 1988; Berthomieu et al., 1992; Roncel et al., 2001). A recent cryo-electron microscopy (cryo-EM) study (Kato et al., 2021) presented a 1.95-Å resolution structural model of the native PSII preparation (PSII-D) from *Thermosynechococcus*, expected to predominantly feature the HP form of Cyt*b*_559_. The bonding distances for the His–Fe heme ligation of Cyt*b*_559_ are about 2.1Å (Figure 2A, Supplementary Table 1). The 1.93-Å resolution cryo-EM structural model of intact PSII preparations from *Synechocystis* (Gisriel et al., 2022), which may predominantly feature the IP form of Cyt*b*_559_, show an apparent elongation in bonding distances (about 2.4Å) for the His–Fe heme ligation of Cyt*b*_559_ (Figure 2B, Supplementary Table 1). In addition, the His–Fe bond of the His 22 residue on the β subunit to the heme is slightly tilted from the heme normal (Supplementary Figure 1). The 2.53-Å resolution cryo-EM structural models of inactive PSII preparations (Apo-PSII-M) of *Synechocystis* (Gisriel et al., 2020), which may predominantly feature the LP form of Cyt*b*_559_ (Ortega et al., 1994; Chiu et al., 2009), show a further increase in bonding distances (about 2.5–2.6 Å) of His–Fe ligation to the heme, tilting of His–Fe bonds, as well as an apparent alteration in the orientations and electrostatic interactions of heme propionate groups of Cyt*b*_559_ (Figure 2C, Supplementary Table 1, Supplementary Figure 1B). These structural changes in His–Fe bonds and heme ligation environments of the Cyt*b*_559_ in inactive PSII are likely induced by conformational changes in the Cyt*b*_559_ α and β subunits associated with loss of extrinsic polypeptides and psbJ (Gisriel et al., 2020).

In addition, striking changes in the His–Fe ligation as well as orientation and interacting environments of the heme propionates of Cyt*b*_559_ were observed in the 2.7-Å resolution cryo-EM structural model of inactive LHCII-PSII supercomplex of *Arabidopsis* (Graça et al., 2021; Figure 2D, Supplementary Table 1). The bonding distances of His-Fe ligations of the heme in Cyt*b*_559_ of inactive PSII of *Arabidopsis* were 3 and 1.9 Å. In addition, a recent Cryo-EM study using the ΔpsbJ mutant of *Thermosynechococcus* reported a structure for the Apo-PSII monomer (Apo-PSII-M) without psbJ (Zabret et al., 2021). The heme-coordination structure of Cyt*b*_559_ in Apo-PSII-M was distorted. The bonding distances of His-Fe ligations of the heme in Cyt*b*_559_ of *T. elongatus* were unevenly elongated from 2.1 Å in the native PSII (Figure 2A) to 2.4 and 2.8Å in Apo-PSII-M (Figure 2E). In addition, a conserved electrostatic interaction between the arginine 18 residue on the beta subunit of Cyt*b*_559_ and a heme propionate was replaced by the arginine 8 residue on the α subunit of Cyt*b*_559_ in the Apo-PSII-M of *Thermosynechococcus*. In contrast, the heme coordination structures of Cyt*b*_559_ in the active PSII-LHCII supercomplex of pea (Su et al., 2017) and the native PSII monomer of *Thermosynechococcus* (Yu et al., 2021) were very similar to that in the native PSII dimer of *Thermosynechococcus* (Supplementary Table 1).

Taken together, the differences in the bonding of His–Fe ligation and the electrostatic environment of the two heme propionate groups of Cyt*b*_559_ may serve as important structural determinants for different redox forms of Cyt*b*_559_ in various PSII preparations. These striking structural changes in the heme ligation environment for inactive PSII is expected to change the hydrophobicity of the heme ligation environment (Krishtalik et al., 1993; Roncel et al., 2003) and may also facilitate the binding of exogenous ligands for superoxide oxidase and reductase activities (Tiwari and Pospíšil, 2009; Pospisil, 2011). Of note, a previous FTIR study reported significant structural changes in the environment of a histidine ligand and a propionic group of the heme between the LP and HP forms of Cyt*b*_559_ (Berthomieu et al., 1992). In addition, previous site-directed mutant results also demonstrated that changes in bonding of His–Fe heme ligation and the electrostatic environment of the heme propionates of Cyt*b*_559_ strongly influenced the ratio of different redox forms of Cyt*b*_559_ in mutant PSII (Hung et al., 2010; Chiu et al., 2013; Guerrero et al., 2014). However, because the peripheral location of Cyt*b*_559_ in the PSII complex may result in uncertainty on heme iron coordination structure (refer to the B values in Supplementary Table 1), structural models at higher resolution may be required to validate whether the variations in the structure of His–Fe ligation are significant in current lower-resolution models of inactive and assembly intermediates of PSII (Figure 2, Supplementary Table 1).

## PsbY Protein is Required for the High Redox Potential Form of Cyt*b*_559_ in *Arabidopsis*

A recent study on ΔpsbY *Arabidopsis* mutants showed that Cyt*b*_559_ was present in only its oxidized LP form in the absence of the PsbY protein (von Sydow et al., 2016). No HP form of Cyt*b*_559_ was found in ΔpsbY *Arabidopsis* mutants. In contrast, wild-type and complement mutant plants contained about 50% of the HP form of Cyt*b*_559_. PsbY was proposed to protect the heme of Cyt*b*_559_ against reducing agents or affect the coordination environment of Cyt*b*_559_, thus leading to changes in its redox properties (von Sydow et al., 2016). The steady-state oxygen evolution activities in mutant plants were comparable to that in wild-type plants under normal light conditions but mutant plants were more susceptible to photoinhibition than the wild type under high light conditions (von Sydow et al., 2016).

## A Thylakoid Membrane-Bound Rubredoxin May Act Together With Cyt*b*_559_ in *De Novo* Assembly and Repair of PSII

A conserved thylakoid membrane-bound rubredoxin (RBD1 in photosynthetic eukaryotes and RubA in cyanobacteria) is required for PSII biogenesis in diverse oxygenic photoautotrophs (Calderon et al., 2013; García-Cerdán et al., 2019; Kiss et al., 2019; Che et al., 2022). RubA-deficient mutant strains of *Synechocystis* were unable to maintain photoautotrophic growth under fluctuating light and showed severe defects in assembly of the heterodimeric D1/D2 reaction center complex (Kiss et al., 2019). A recent study on RBD1 mutants using *C. reinhardtii* indicated that the transmembrane domain of RBD1 is essential for *de novo* PSII assembly, and that its rubredoxin domain is involved in PSII repair (García-Cerdán et al., 2019). In addition, the rubredoxin domain of RBD1 (and RubA) is exposed to the cytoplasm and exhibits a redox midpoint potential of +114 mV. Reduction of RBD1 content can be mediated by ferredoxin-NADP+ reductase *in vitro* (García-Cerdán et al., 2019). These results suggest that RBD1 (and RubA) may act together with Cyt*b*_559_ to protect the intermediates of PSII reaction center complexes against photooxidative damage during *de novo* assembly and repair (García-Cerdán et al., 2019; Kiss et al., 2019).

## Psb28 Protein Binds to Cyt*b*_559_ in the RC47 Complex During the Assembly of PSII

A recent study that conducted chemical cross-linking combined with mass spectrometry predicted the location of Psb28 to be in close proximity to the N-terminal domain of the Cyt*b*_559_ protein (Weisz et al., 2017). In addition, this study proposed a protective role for Psb28, whereby it blocks electron transport in the acceptor side of PSII to protect the RC47 complex against excess photodamage during the assembly process. Another recent study, which conducted cryo-EM, solved the structure of the PSII assembly intermediate from a deletion strain of the psbJ of *T. elongatus* (Zabret et al., 2021). The deletion of PsbJ stalled PSII assembly at a specific transition and induced the accumulation of assembly factors Psb27 and Psb28. The cryo-EM map (2.94Å) of PSII-I (for the PSII intermediate) provided a snapshot of the attachment of the CP43 module to the pre-assembled RC47 complex (Zabret et al., 2021). This PSII-I contains 3 assembly factors (Psb27, Psb28, and Psb34). In the structure of PSII-I, Psb28 binds on cytosolic faces of the D1 and D2 subunits, directly above the Q_B_ binding site. The binding of Psb28 induced large conformational changes at the PSII acceptor sideof the RC47 complex, which distorted the Q_B_binding pocket and replaced the bicarbonate ligand of non-heme iron with glutamate. This distinct structural motif is also present in reaction centers of non-oxygenic photosynthetic bacteria (Zabret et al., 2021). These results reveal the structural and functional roles of psb28 in protecting the RC47 complex against damage during PSII assembly. Furthermore, the structure of the heme coordination of Cyt*b*_559_ in PSII-I was significantly distorted. The bonding distances for axial heme ligations of Cyt*b*_559_ were elongated (2.6 and 2.8Å), and the electrostatic interactions between two propionate groups of the heme and 2 conserved arginine residues were weakened or broken (Figure 2F, Supplementary Table 1).

## The Q_C_ Site May Be Involved in Modulating Short-Term Light Responses in PSII of Cyanobacteria

A previous study on the 2.9-Å resolution PSII crystal structure revealed the binding of a PQ molecule, Q_C_, in a hydrophobic cavity near Cyt*b*_559_ (Guskov et al., 2009). Previous studies proposed that the Q_C_ site may be involved in exchange of PQ/plastoquinol (PQH_2_) on the Q_B_ site from the pool (Guskov et al., 2009; Müh et al., 2012) or in modulating the redox potential and reactivity of Cyt*b*_559_ (Kruk and Strzałka, 1999; Kruk and Strzalka, 2001; Kaminskaya et al., 2007a,b; Bondarava et al., 2010). However, the binding of PQ to the Q_C_ site appeared to be weak or transient (Koji and Takumi, 2014; Van Eerden et al., 2017), and the Q_C_ molecule was not detected in recent high-resolution crystal structure models of native PSII from *T. vulcanus* (Umena et al., 2011; Suga et al., 2015).

Several Q_C_-site *Synechocystis* mutant strains (e.g., S28Aβ, V32Fβ, and A16FJ) showed significantly higher photosynthesis growth rate and biomass accumulation than wild-type strains (Huang et al., 2016, 2018). In addition, the ratios of redox potential forms of Cyt*b*_559_ for Q_C_-site mutant PSII core complexes were similar to those for the wild type. Furthermore, Q_C_-site mutant cells had distinct effects on short-term light responses (state transition and blue light-inducing non-photochemical quenching) (Huang et al., 2016, 2018). Taken together, the results suggest that the mutations on the Q_C_ site of PSII may modulate short-term light adaptations of the photosynthetic apparatus in *Synechocystis*.

## Conclusions and Perspectives

Recent mutagenesis studies combined with high-resolution protein crystallography and cryo-EM structural analysis as well as functional genomics and biochemical analysis have greatly advanced our understanding of the structural and functional roles of Cyt*b*_559_ in the assembly, proper function, and photoprotection of PSII. Studies have revealed possible structural determinants for different redox forms of Cyt*b*_559_ in various PSII preparations. In addition, several assembly factors and protein subunits may act together with Cyt*b*_559_ to protect the intermediates of PSII reaction center complexes during *de novo* assembly and repair. These integrated approaches may lead to the final proof of the molecular mechanisms of Cyt*b*_559_ in PSII.

## Author Contributions

Y-FC and H-AC wrote the article. H-AC acquired the funding and supervised the project. Both authors read and approved the manuscript.

## Funding

This study was supported by Academia Sinica for H-AC.

## Conflict of Interest

The authors declare that the research was conducted in the absence of any commercial or financial relationships that could be construed as a potential conflict of interest.

## Publisher's Note

All claims expressed in this article are solely those of the authors and do not necessarily represent those of their affiliated organizations, or those of the publisher, the editors and the reviewers. Any product that may be evaluated in this article, or claim that may be made by its manufacturer, is not guaranteed or endorsed by the publisher.

## Abbreviations

Car: β-carotene
Cryo-EM: cryo-electron microscopy
Cyt*b*_559_: cytochrome *b*_559_
EPR: electron paramagnetic resonance
LHCII: light-harvesting complex II
HP: high potential
IP: intermediate potential
LP: low potential
PQ: plastoquinone
PQH_2_: plastoquinol
PSII: photosystem II
Q_B_: the secondary quinone electron acceptor in PSII
Q_C_: the third plastoquinone-binding site in PSII.
