# Supplementary material for: New Structural and Mechanistic Insights Into Functional Roles of Cytochrome b559 in Photosystem II
Source: Front Plant Sci. 2022 Jun 8;13:914922. doi: 10.3389/fpls.2022.914922 (PMC9214863; doi:10.3389/fpls.2022.914922)
Supplement: Supplementary file 1 [file Table_1.pdf]

Supplemental Table 1 Properties of the heme-ligation structures of Cyt b_{559} in native, inactive and assembly intermediates of photosystem II (PSII) from cyanobacteria and plants by high-resolution cryo-electron microscopy structural studies.

**Species** Resolution (Overall B-value)	**PDB**	**PSII type**	**Bonding distances for His–Fe ligations (B-values of the two His and Fe atoms)**	**Propionate electrostatic interactions**	**Reference**
Thermosynechococcus vulcanus 2.08Å (low dosage) (12.4)	7D1U	Native PSII-D (with all subunits)	2.1 and 2.0 Å (26.8, 20.0 and 21.6)	Normal	Kato et al., 2021
Thermosynechococcus vulcanus 1.95Å (high dosage) (11.9)	7D1T	Native PSII-D (with all subunits)	2.0 and 2.1 Å (23.5, 18.2 and 19.0)	Normal	Kato et al., 2021
Thermosynechococcus elongatus 2.94Å (108)	7NHP	PSII-I (with Psb27, Psb28 and Psb34 but lack of psbJ and extrinsic proteins)	2.6 and 2.8 Å (169, 142, and 160)	Altered	Zabret et al., 2021
Thermosynechococcus elongatus 2.82Å (118)	7NHO	Inactive PSII-M (lack of psbJ)	2.4 and 2.9 Å (165, 147 and 158)	Altered	Zabret et al., 2021
Thermosynechococcus vulcanus 2.78Å (21.4)	7EDA	Intact PSII-M (with all subunits)	2.1 and 2.1 Å (24.9, 20.6 and 23.8)	Normal	Yu et al., 2021
Synechocystis PCC6803 2.01Å (low dosage)	7RCV	Intact PSII-D (with all subunits)	2.5 and 2.4 Å (15.5, 13.7 and 15.5)	Normal	Gisriel et al., 2022

(6.6)					
Synechocystis PCC6803 1.93Å (high dosage) (3.8)	7N8O	Intact PSII-D (with all subunits)	2.4 and 2.3 Å (8.3, 9.4 and 8.6)	Normal	Gisriel et al., 2022
Synechocystis PCC6803 2.58Å (35.0)	6WJ6	Inactive Apo-PSII-M (without psbJ, psbY, psbZ and extrinsic proteins)	2.6 and 2.6 Å (71.7, 63.4 and 69.5)	Altered	Gisriel et al., 2020
Pea 2.7Å (118)	5XNL	Active, Stacked C2S2M2 PSII-LHCII supercomplex	2.1 and 2.0 Å (50.7 and 52.4 and 48.5)	Normal	Su et al., 2017
Spinach 3.2Å (78.1)	3JCU	Active PSII-LHCII supercomplex (Without psbY and psbR)	2.6 and 2.5 Å (156, 157 and 111)	Altered	Wei et al., 2015
Arabidopsis 2.79Å (87.0)	7OUI	Inactive PSII-LHCII supercomplex	3.0 and 1.9 Å (73.5, 60.0 and 67.9)	Altered	Graca et al., 2021

The overall b-values were calculated by using the service at the following website https://swift.cmbi.umcn.nl/servers/html/listavb.html
