# Supplementary material for: New Structural and Mechanistic Insights Into Functional Roles of Cytochrome b559 in Photosystem II
Source: Front Plant Sci. 2022 Jun 8;13:914922. doi: 10.3389/fpls.2022.914922 (PMC9214863; doi:10.3389/fpls.2022.914922)
Supplement: Supplementary file 2 [file Figure_1.pdf]

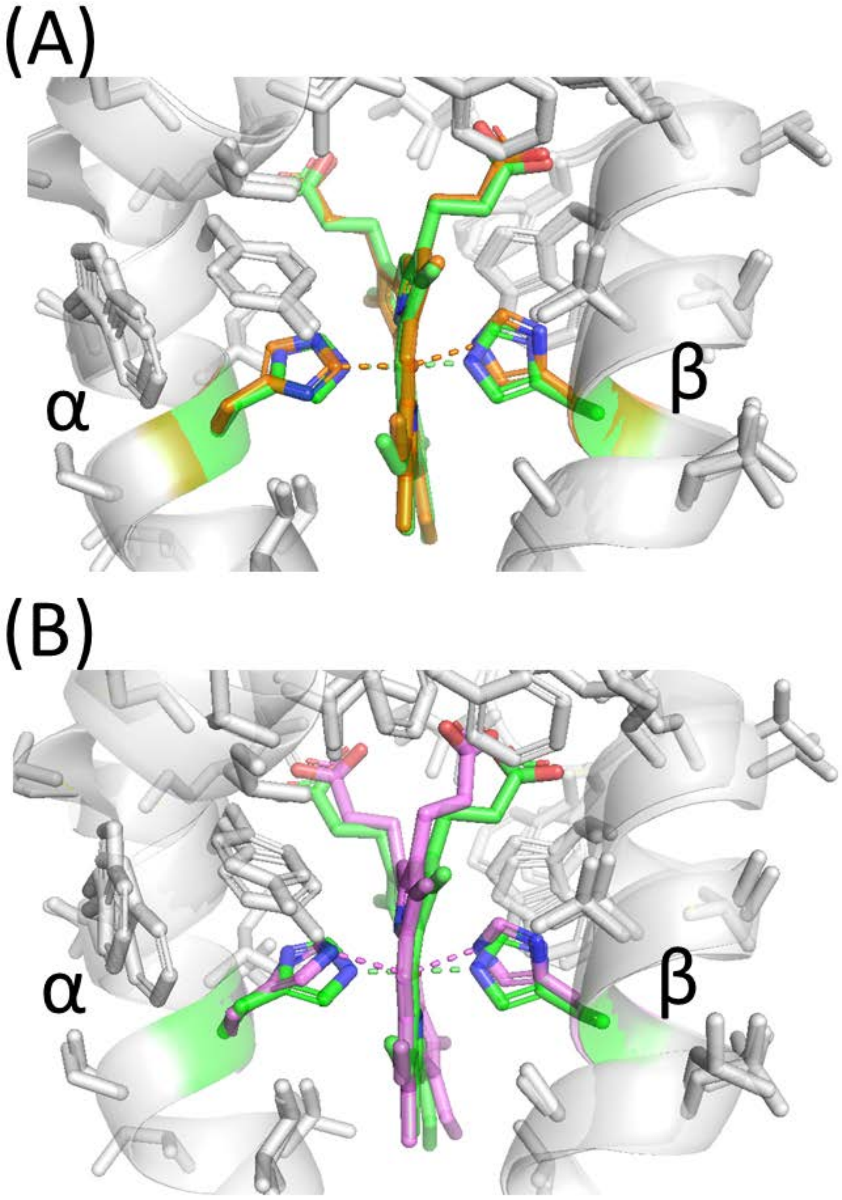

Supplemental Figure 1. Comparison of the heme-ligation structures of Cyt b_{559} in Cryo-EM structural models of different cyanobacterial PSIIs. (A) Overlay of native PSII-D model from *Thermosynechococcus* (PDB 7D1U, green) with intact PSII-D model from *Synechocystis* (PDB 7RCV, orange). (B) Overlay of native PSII-D model from *Thermosynechococcus* (PDB 7D1U, green) with inactive Apo-PSII monomer from *Synechocystis* (PDB 6WJ6, pink).
